# Supplementary figures and images for: Transcriptomic signatures of WNT-driven pathways and granulosa cell-oocyte interactions during primordial follicle activation
Source: PLoS One. 2024 Oct 23;19(10):e0311978. doi: 10.1371/journal.pone.0311978 (PMC11498688; doi:10.1371/journal.pone.0311978)

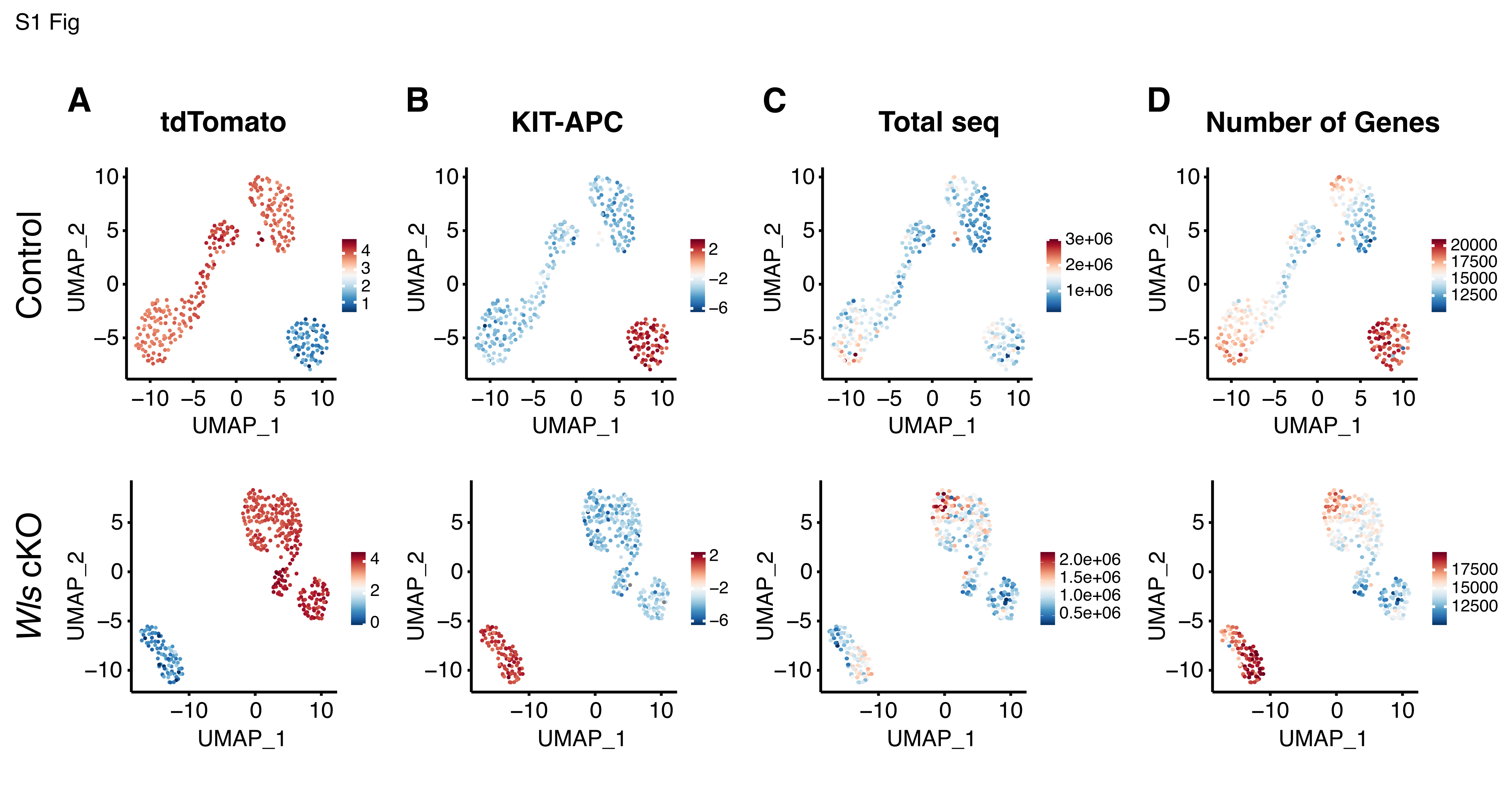

Supplement: S1 Fig — UMAP plots showing tdTomato-expressing somatic cells (A), the KIT-APC positive oocyte population (B), the total number of mapped reads (total seq) (C), and the number of detected genes (number of genes) (D). The color scale represents the gene expression level scaled by the Z-score. (TIF) [file pone.0311978.s001.tif]

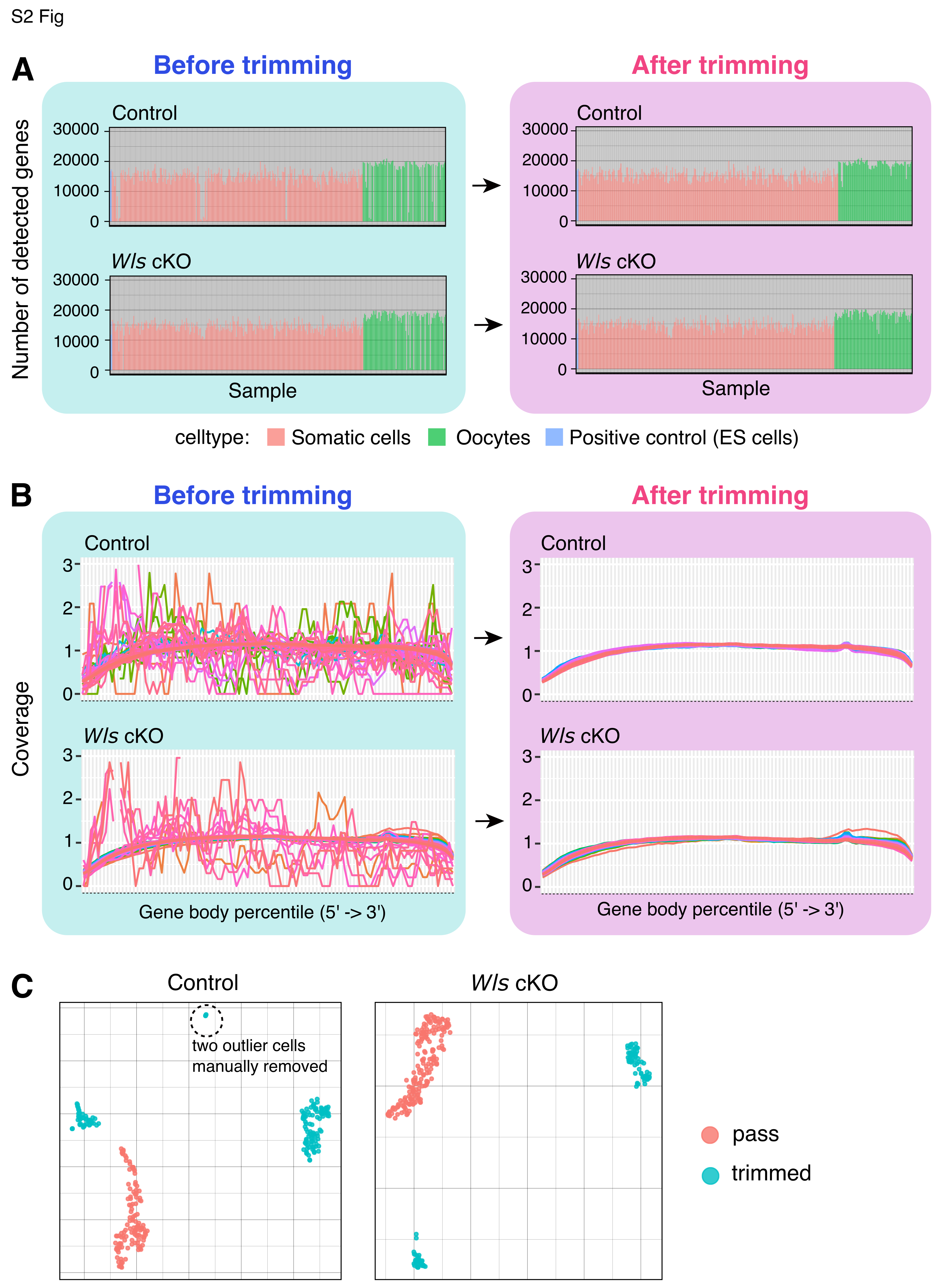

Supplement: S2 Fig — (A) Number of detected genes or (B) Gene body coverage before and after trimming of the single-cell RNA sequencing data, showing the normalized distribution of read coverage across the length of genes. Trimming was performed following MultiQC guidelines. (C) UMAP plot showing two outlier cells that were identified and excluded from the control group of somatic cells. (TIF) [file pone.0311978.s002.tif]

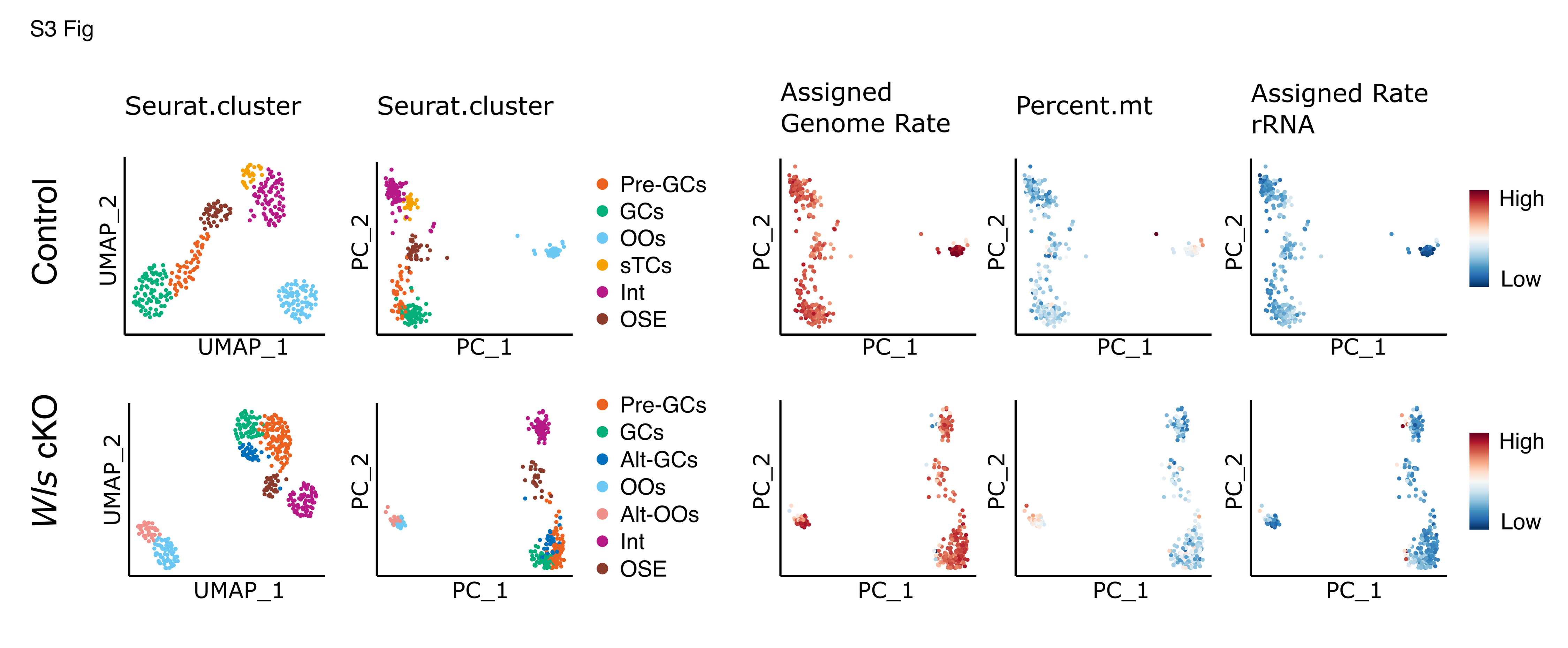

Supplement: S3 Fig — PCA plots with overlaid key quality metrics post-QC filtering, including the assigned genome rate, mitochondrial gene mapping rate, and rRNA mapping rate. (TIF) [file pone.0311978.s003.tif]

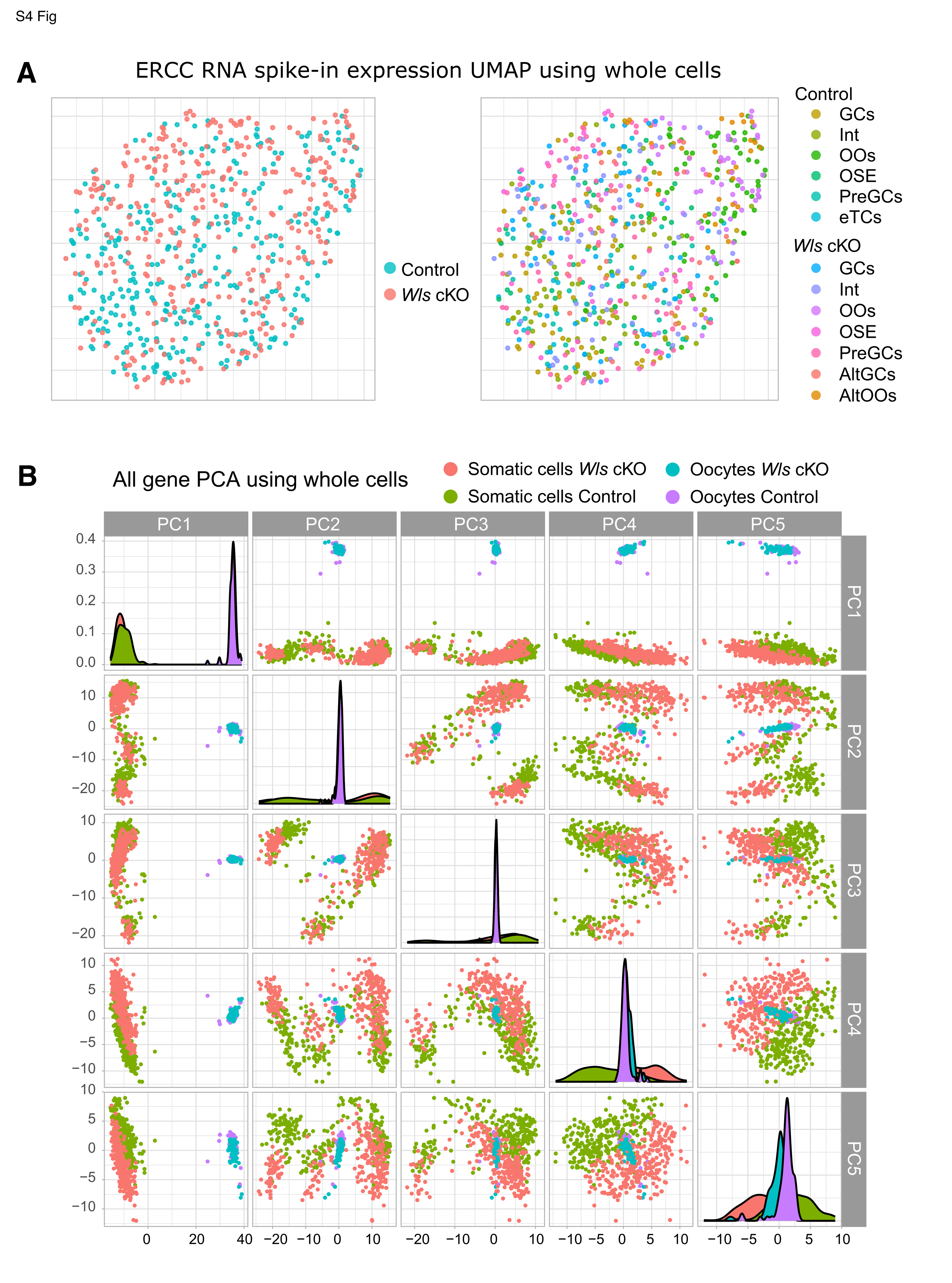

Supplement: S4 Fig — (A) UMAP plots of ERCC RNA spike-in transcripts showing highly mixed distribution regardless of genotype (left) and cell type (right). (B) PCA plots based on expression of all genes showing the first three principal components were not associated with genotype. (TIF) [file pone.0311978.s004.tif]

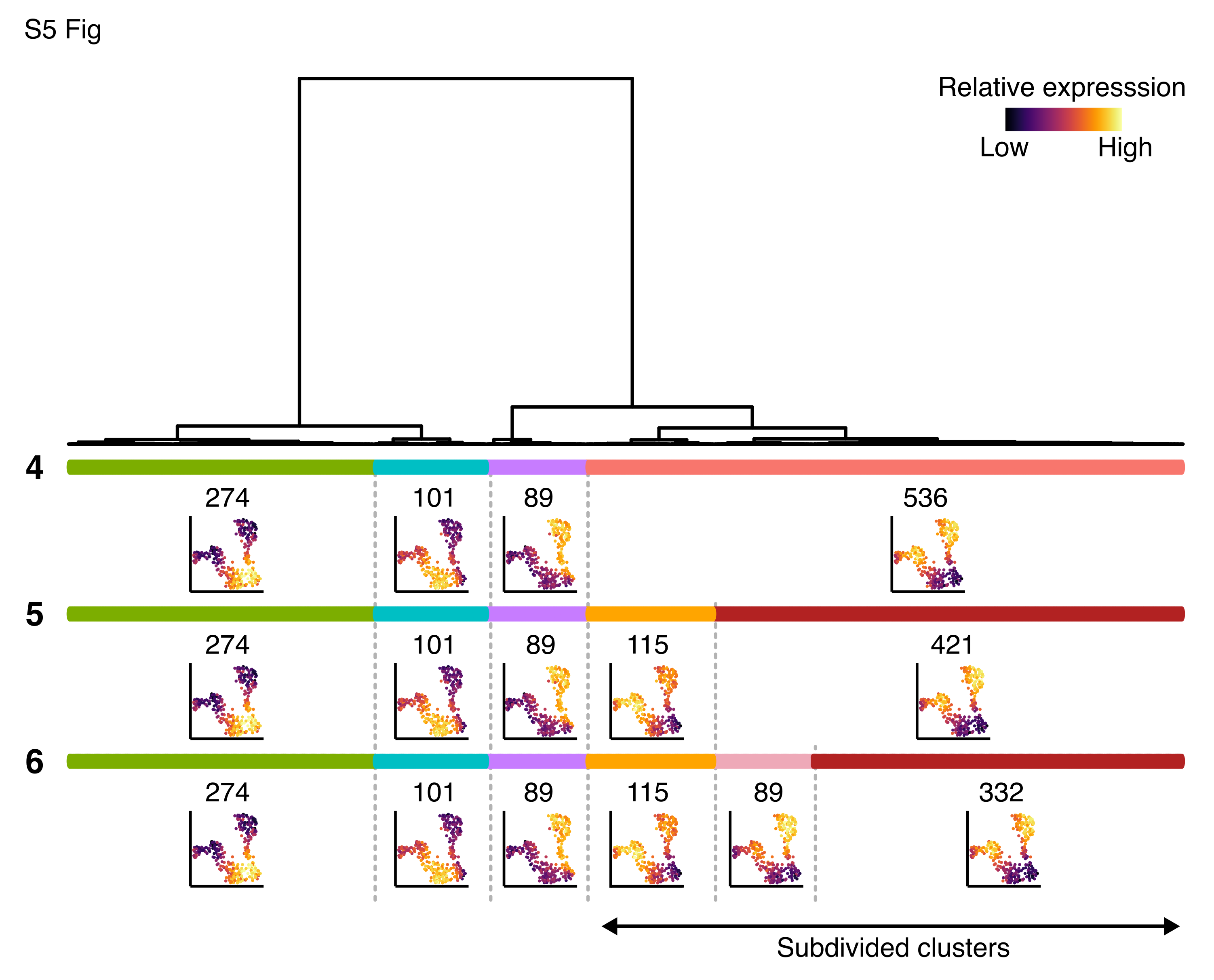

Supplement: S5 Fig — Diagram of hierarchical clustering based on the kinetics of DEGs obtained by the singleCellHaystack method in the GC population. Color bars represent the clustering result when the dendrogram is cut into 4–6 clusters. The number to the left of each bar indicates the number of clusters. The average expression levels of the genes in each cluster were projected onto UMAP plots below the corresponding bars. More clusters lead to a greater subdivision of the main DEG cluster. (TIF) [file pone.0311978.s005.tif]

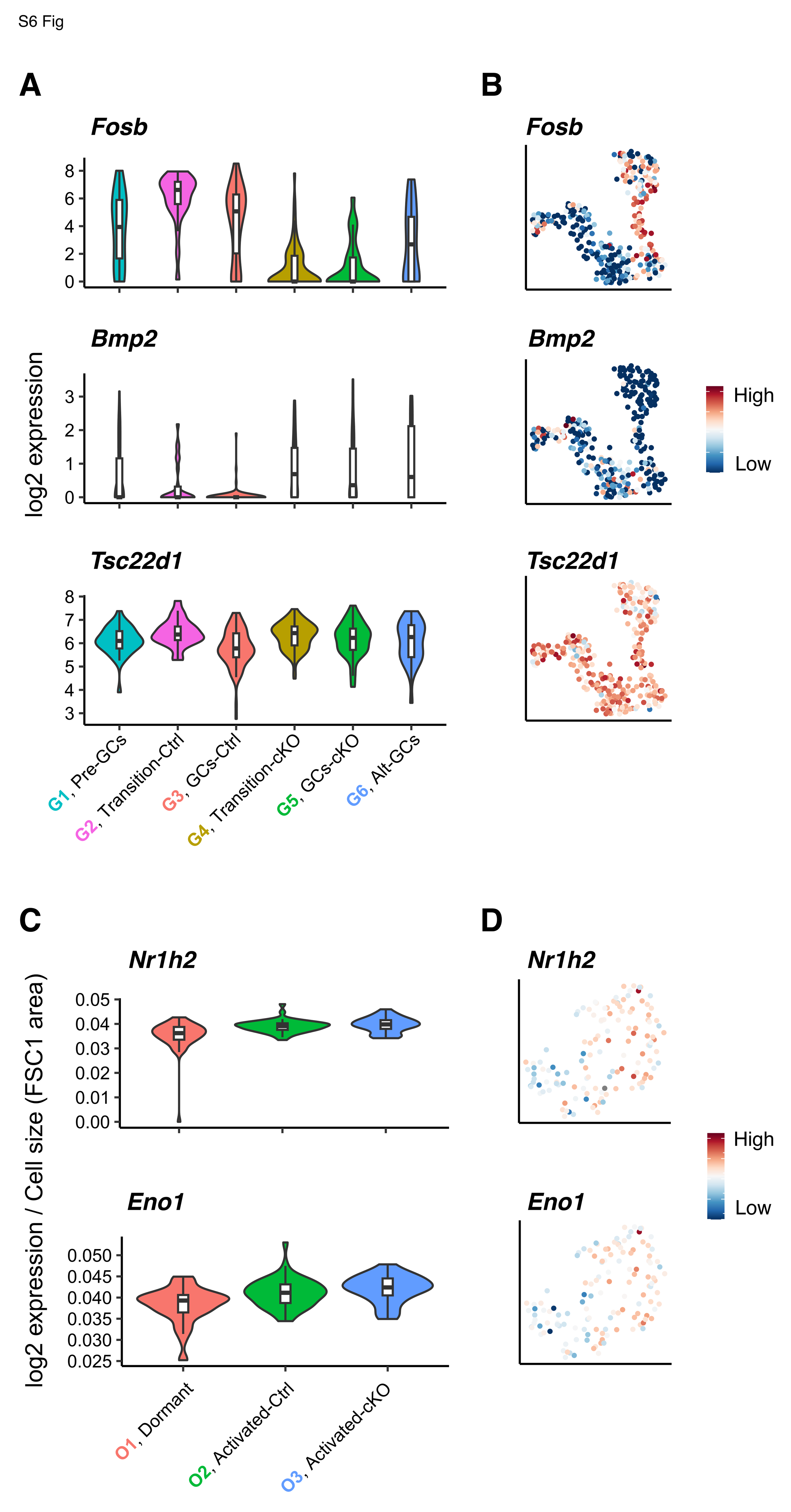

Supplement: S6 Fig — (A, B) Gene expression validation in granulosa cells (GCs) measured by single-cell RT-qPCR (scRT-qPCR). The expression levels of Fosb, Bmp2, and Tsc22d were analyzed. (A) Violin plots depict gene expression levels across GC subclusters, while (B) UMAP projections show the spatial distribution patterns of these genes. (C, D) scRT-qPCR validation of gene expression in oocytes. The expression levels of Nr1h2 and Eno1 were analyzed, with (C) showing violin plots and (D) displaying UMAP projections to compare expression across oocyte subclusters. (TIF) [file pone.0311978.s006.tif]

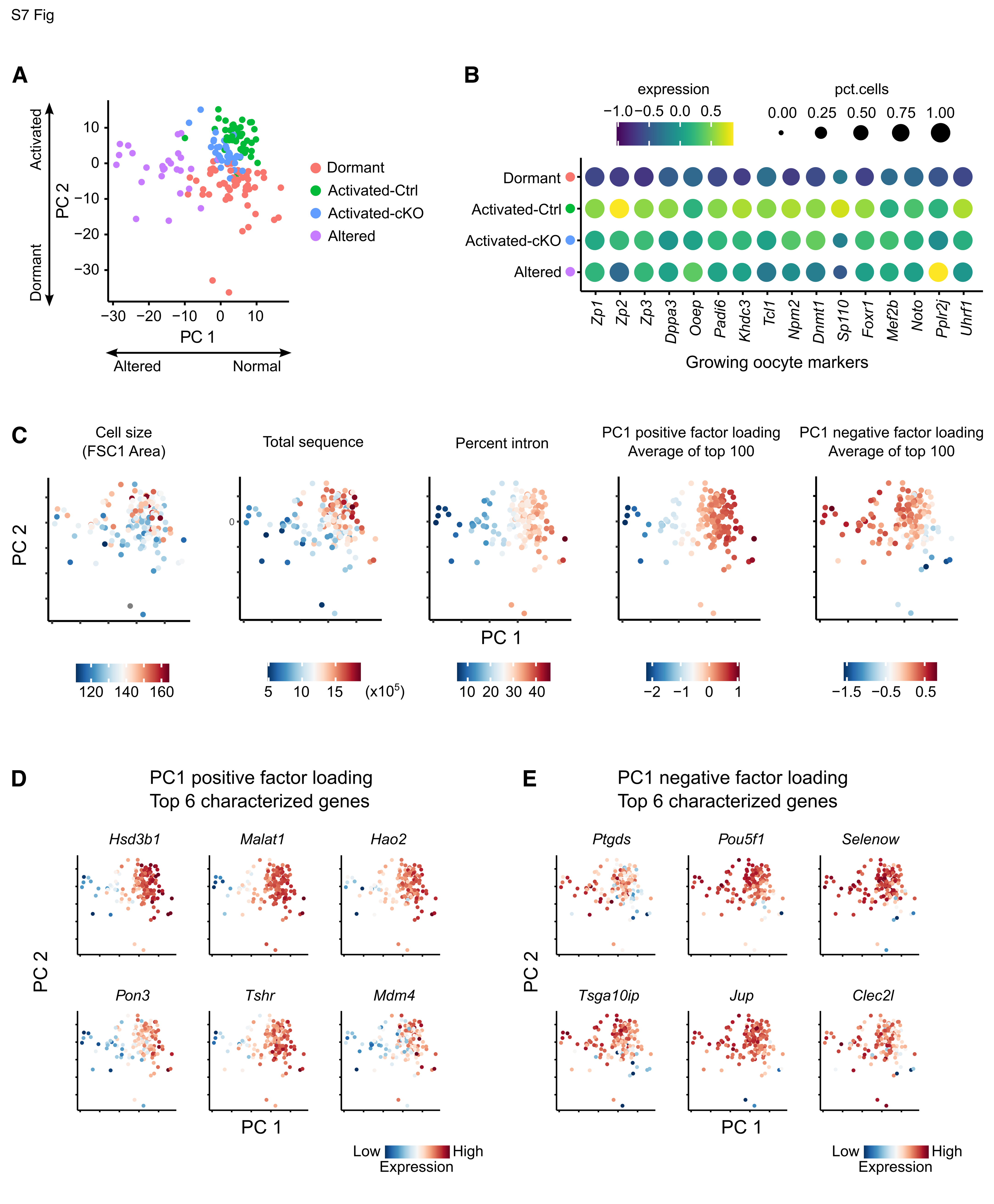

Supplement: S7 Fig — (A) PCA plots of oocytes. Different colors correspond to subclusters of oocytes. The principal components on the axes capture the major sources of gene expression variation, with their respective contributions indicated in the axis labels. (B) Dot plot showing the expression of growing oocyte markers. The dot size represents the ratio of cells expressing a specific marker (TPM > 1), while the color indicates the average gene expression level scaled by the Z-score. (C) UMAP plots showing cell size, the total number of mapped reads (total sequence), the ratio of intronic reads (percent intron), the PC1 positive factor loading and the PC1 negative factor loading. (D) UMAP plots showing the top 6 characterized genes of PC1 positive factor loading. (E) UMAP plots showing the top 6 characterized genes of PC1 negative factor loading. (TIF) [file pone.0311978.s007.tif]

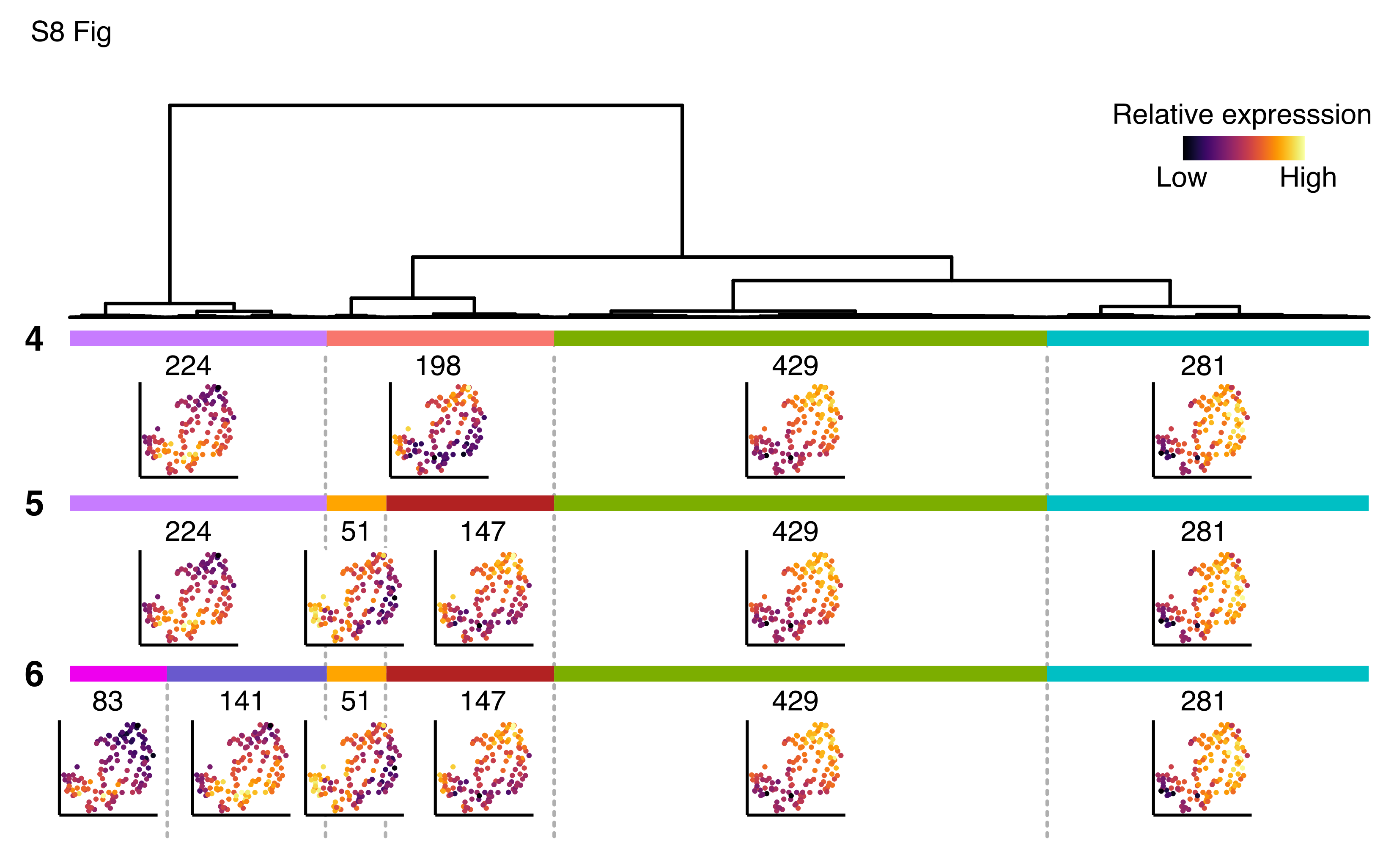

Supplement: S8 Fig — Diagram of hierarchical clustering based on the kinetics of DEGs obtained by the singleCellHaystack method in the oocyte population. Color bars represent the clustering result when the dendrogram is cut into 4–6 clusters. The number on the left of each bar indicates the number of clusters. The average expression level of the genes in each cluster were projected onto UMAP plots below the corresponding bars. (TIF) [file pone.0311978.s008.tif]
