# Supplementary material for: Transcriptomic signatures of WNT-driven pathways and granulosa cell-oocyte interactions during primordial follicle activation
Source: PLoS One. 2024 Oct 23;19(10):e0311978. doi: 10.1371/journal.pone.0311978 (PMC11498688; doi:10.1371/journal.pone.0311978)
Supplement: Attachment — Submitted filename: review-0321.docx [file pone.0311978.s019.docx]

The manuscript investigates the transcriptional changes of granulosa cells and oocytes during Primordial follicle activation (PFA) using Wntless (Wls) conditional knockout (cKO) mice, emphasizing the impact of the role of the WNT pathway in defining granulosa cell identity and their interactions with oocytes. This work contributes to our understanding of the mechanisms underlying the synchronous dormancy and activation of granulosa cells and oocytes during the PFA process. However, there are still some significant issues in the manuscript that require substantial revisions.

Major

This study utilizes the RamDA-seq, a full-length total RNA-sequencing method at the single-cell level, to examine GCs and oocytes. However, The choice of this method, along with its advantages over traditional single-cell RNA sequencing techniques, is not adequately justified or elaborated upon in the text. Does "full-length" sequencing imply the ability to analyze expression differences of isoforms between different cell clusters in control and Wls cKO mice?

The data analysis provided seems to lack depth. The absence of fundamental quality control measures and adjustments raises doubts about the reliability of the conclusions made.

The referencing order of the figures in the results section of the manuscript appears to be disorganized. Some figures are not well-associated with the corresponding text they are referenced to.

Minor

L38 The manuscript suggests, "The formation of primordial follicles occurs shortly after birth in mice," however, the process of primordial follicle formation actually begins shortly before birth, which also contradicts the statement in L96-97.

L54-57 FOXO3, GDF9, and other gene names should be italicized.

L79-85 The statements about what you have done following questions 2 and 3 might be better placed in a different context.

L166 In Figure 2, the colors representing the same cell types in both the control and Wls cKO groups should be consistent. This would allow for a clear visualization of the differences between the control and Wls cKO mice.

L177 Was the batch effect considered when integrating the single-cell data from Wls cKO and Ctrl groups.

L312-314 What is the reason for the notable difference in IRX3 expression between control and Wls cKO as shown in Figures 4E and 4F?
