# Supplementary material for: Transcriptomic signatures of WNT-driven pathways and granulosa cell-oocyte interactions during primordial follicle activation
Source: PLoS One. 2024 Oct 23;19(10):e0311978. doi: 10.1371/journal.pone.0311978 (PMC11498688; doi:10.1371/journal.pone.0311978)
Supplement: Attachment — Submitted filename: Comments for Manuscript PONE-D-24-08138.docx [file pone.0311978.s020.docx]

**Reviewer`s Comments for Manuscript PONE-D-24-08138**

Elucidation of the role and molecular mechanism of WNT-driven pathways in GC maturation, primordial follicle activation and folliculogenesis is important for understanding ovarian physiology and female reproductive biology. Whilst interesting, there are several concerns that need to be addressed or clarified.

1. Line 541-575, in the"single cells sorting", how authors to ensure only GCs were separated from the follicles? and what method was utilized to further identify the cells that were used for transcritomic analysis?

2. Line 166-172, Line 577-586, in the “Quality control” of scRNA-seq, principal component analysis (PCA) related results that can demonstrated the most important correlation or repeatability between the “single cells” samples should be supplemented.

3. To facilitate readers to understand the critical role of the WNT-signaling pathway and its molecular signature in regulating normal follicular development, the newly revealed key genes or signaling pathways involved in crosstalk with GCs, GC maturation and differentiation, and oocyte growth should be clearly indicated or provided, and one or two of them are suggested to be further verified.
