# Supplementary material for: Transcriptomic signatures of WNT-driven pathways and granulosa cell-oocyte interactions during primordial follicle activation
Source: PLoS One. 2024 Oct 23;19(10):e0311978. doi: 10.1371/journal.pone.0311978 (PMC11498688; doi:10.1371/journal.pone.0311978)
Supplement: Attachment — Submitted filename: Response_to_Reviewers.docx [file pone.0311978.s021.docx]

We are grateful to the reviewers for their valuable time and insightful comments on our paper. We have carefully addressed all of the points raised and have made the following revisions to enhance the quality and clarity of our manuscript:

- A detailed overview of the quality control procedures has been provided.
- Analyses were conducted to confirm that batch effects were minimal.
- qPCR was performed to validate key gene expressions, confirming the scRNA-seq results.
- Immunofluorescent staining was repeated using PD7 samples, and the results have been updated accordingly.

The corresponding sections in the main text have been revised to reflect these updates. Below, we provide point-by-point responses to the reviewers' comments.

**Reviewer #1**: The manuscript investigates the transcriptional changes of granulosa cells and oocytes during Primordial follicle activation (PFA) using Wntless (Wls) conditional knockout (cKO) mice, emphasizing the impact of the role of the WNT pathway in defining granulosa cell identity and their interactions with oocytes. This work contributes to our understanding of the mechanisms underlying the synchronous dormancy and activation of granulosa cells and oocytes during the PFA process. However, there are still some significant issues in the manuscript that require substantial revisions.

**Major**

1. This study utilizes the RamDA-seq, a full-length total RNA-sequencing method at the single-cell level, to examine GCs and oocytes. However, The choice of this method, along with its advantages over traditional single-cell RNA sequencing techniques, is not adequately justified or elaborated upon in the text. Does “full-length” sequencing imply the ability to analyze expression differences of isoforms between different cell clusters in control and Wls cKO mice?

**Response 1-1.** Based on our preliminary flow cytometry analysis, the ratio of oocytes to GCs in our sample was estimated to be approximately 1:300. To achieve sufficient sequencing coverage for oocytes, which required higher read depth per cell, it was necessary to enrich these cells through cell sorting. Additionally, given that our experiment involved triple-mutant mice, the total number of cells available for analysis was limited. This constraint led us to select a plate-based method. Among the available plate-based methods, we chose RamDA-seq because it is highly sensitive and has a proven track record in oocyte analysis [20]. Our choice of RamDA-seq was not driven by a specific need to detect full-length RNA in this study.

Furthermore, oocytes are large cells with a diameter of at least 20 μm. In contrast, microfluidics-based methods are typically optimized for smaller cells with diameters of less than 10 μm, including GCs. Therefore, we reasoned that using a microfluidics-based approach would be challenging for handling a mixture of such differently-sized cells.

We also considered the RNA content of each cell type. Oocytes contain significantly more RNA than GCs, and droplet-based methods could result in insufficient read depth for GCs. In fact, we observed a difference of more than two cycles in scRT-qPCR for Gapdh between oocytes and GCs, and about a fourfold difference in library yield. By using the plate-based RamDA-seq method, we were able to separate oocytes and GCs through cell sorting and adjust the molar ratio when pooling libraries, ensuring sufficient read depth for both cell types. We have added an explanation at L114-117, clarifying why RamDA-seq was chosen.

2. The data analysis provided seems to lack depth. The absence of fundamental quality control measures and adjustments raises doubts about the reliability of the conclusions made.

**Response 1-2.** Thank you for your feedback. We understand the importance of quality control in ensuring the reliability of our single-cell RNA sequencing data. In response to your concerns, we have significantly expanded the quality control section of our manuscript in the Results section (L 124–128). We have included several key quality control metrics to enhance the transparency and rigor of our data evaluation process.

We have added detailed figures that display gene body coverage and the proportion of reads aligned to the reference genome before and after trimming outlier cells in our RamDA-seq analysis (S2 Fig). These figures illustrate that our trimming process effectively removed low-quality cells while preserving the overall integrity of the dataset. Additionally, we performed a Principal Component Analysis (PCA) that integrates key quality metrics such as the assigned genome rate, mitochondrial gene content, and rRNA percentage (S3 Fig). This PCA plot visually demonstrates the clustering of high-quality cells and highlights the separation of outliers, supporting the robustness of our data processing pipeline. We believe these revisions substantially strengthen the depth and reliability of our analysis, providing greater confidence in the validity of our findings.

3. The referencing order of the figures in the results section of the manuscript appears to be disorganized. Some figures are not well-associated with the corresponding text they are referenced to.

**Response 1-3.** Thank you for your valuable feedback. We acknowledge that the referencing order of Fig 3 may have been unclear. To address this, we have carefully revised the manuscript and added explanations in L180–184 to improve the flow and ensure that Fig 3 is more clearly linked to the corresponding text. We hope that these revisions address your concerns.

**Minor**
L38 The manuscript suggests, “The formation of primordial follicles occurs shortly after birth in mice,” however, the process of primordial follicle formation actually begins shortly before birth, which also contradicts the statement in L96-97.

**Response 1-4.** We have revised L39 to accurately state that primordial follicle formation begins shortly before birth (perinatally) in mice, ensuring consistency with the statement in L81.

L54-57 FOXO3, GDF9, and other gene names should be italicized.

**Response 1-5.** We understand that gene names should be italicized when referring to the gene itself, while protein names should not be italicized. In the specific lines you mentioned (L54-57), FOXO3 and GDF9 were used to refer to proteins rather than genes. We have reviewed the manuscript to ensure that gene names are italicized only when they clearly refer to the gene itself. We believe the current usage is consistent with standard conventions.

L79-85 The statements about what you have done following questions 2 and 3 might be better placed in a different context.

**Response 1-6.** Thank you for your suggestion. We have moved the statements regarding questions from the Introduction to the Discussion section (L431-433, 448-459, and 484-486). Additionally, we have removed redundant content and adjusted the logical flow of the manuscript to enhance clarity. We hope these revisions address your concern.

L166 In Figure 2, the colors representing the same cell types in both the control and Wls cKO groups should be consistent. This would allow for a clear visualization of the differences between the control and Wls cKO mice.

**Response 1-7.** We have revised Fig 2A-D to ensure consistent color representation for the same cell types in both the control and *Wls* cKO groups. We have selected color schemes that are colorblind-friendly to enhance clarity for all readers.

L177 Was the batch effect considered when integrating the single-cell data from Wls cKO and Ctrl groups.

**Response 1-8.** Thank you for raising this important point. In our analysis, we did not account for batch effects when integrating the single-cell data from the *Wls* cKO and Ctrl groups. This approach was chosen to preserve biological effects and maintain data integrity, as there are no conditions that are strictly identical between the control and *Wls* cKO groups. Furthermore, the original RamDA-seq paper carefully addresses batch effects and demonstrates that the method is highly precise, with minimal batch effect influence [19]. Indeed, potential batch effects during library preparation were negligible based on UMAP clustering analysis using exogenous ERCC RNA spike-in transcripts, which showed a highly mixed genotype-independent distribution (S4A Fig). In addition, PCA for whole cells showed that the major PC axes were not associated with batch (genotype) (S4B Fig), although detailed exploration found minor possible batch effects around PC4 and PC5. Overall, these data support our data integration without normalization for batch effects to avoid loss of biological information as mentioned above. A description regarding batch effects has been added in L170-176 of the manuscript to further clarify this point.

L312-314 What is the reason for the notable difference in IRX3 expression between control and Wls cKO as shown in Figures 4E and 4F?

**Response 1-9.** We apologize for any confusion regarding the expression of IRX3. It appears that there may have been a misunderstanding. The data in Fig 4E and 4F actually show similar trends in IRX3 expression between the control and *Wls* cKO groups. We clarified this in the manuscript at L308-309 to ensure the consistency of IRX3 expression between the two groups.

**Reviewer #2**: No concerns/conflicts of interest to address. No additional comments for the author, including concerns about dual publication, research ethics, or publication ethics. I agree with the above statements pertaining to questions 1-4.

**Reviewer #3**: In this manuscript, differences between ovarian granulosa cells and oocytes of wild-type and Wls cKO mice were analyzed at the single cell transcription level. The significant role of an autocrine function of WNT signaling in pre-GCs during primordial follicles activation, and the gene characteristics related to oocyte dormancy and activation were identified. However, the authors does not give clear conclusions on the three problems originally mentioned in the paper. This description should be carefully reviewed. Also, there are specific comments need to be addressed.

**Response 3-0.** Thank you for your valuable suggestion. We have relocated the statements concerning the questions from the Introduction to the Discussion section (L431-433, 448-459, and 484-486). Furthermore, we have eliminated repetitive content and refined the logical structure of the manuscript to improve clarity. We hope these changes resolve your concern.

Specific Comments:
**Major Issues**:
1. In Fig 2A, provide an explanation for why are there two different clusters of GC and oocyte in Wls cKO mice? How are altered granulosa cells and oocytes defined?

**Response 3-1.** In Fig 2A, the two distinct clusters of GCs and oocytes in *Wls* cKO mice were identified through unsupervised clustering analysis. The clustering algorithm used takes into account the transcriptional profiles of individual cells, leading to the separation of cells into distinct groups based on their gene expression patterns.

Altered Granulosa Cells (Alt-GC): We have defined the Alt-GC cluster based on its proximity to the normal GC cluster, yet with noticeably lower expression levels of general GC markers such as *Amh* and *Inhbb*. Despite their proximity, the distinct gene expression profile of this cluster suggests a unique subpopulation of GCs that may be undergoing pathological changes in the *Wls* cKO mice.

Altered Oocytes (Alt-Oo): As described in the manuscript, the Alt-Oo cluster is characterized by high expression of general oocyte markers such as *Zp3* and *Gdf9*, but these cells also display significantly lower quality control metrics, including reduced ratios of reads mapping to the intronic region. These features suggest that the Alt-Oo cells represent a subpopulation of oocytes that are transcriptionally inactive, possibly compromised in their functionality or viability.

2. In Fig 3A, there is almost no overlap in the distribution of UMAP between the GCs of wild type and Wls cKO mice, and it looks like the data were used ‘merge’ function rather than the ‘integrate’ function. Please include the distribution result of UMAP and PCA after direct merge and the PCA result after integrate.

**Response 3-2.** Thank you for your comment. In our analysis, we intentionally used the ‘merge’ function rather than the ‘integrate’ function. We did not account for batch effects when integrating the single-cell data from the *Wls* cKO and Ctrl groups. This approach was chosen to preserve biological effects and maintain data integrity, as there are no conditions that are strictly identical between the control and *Wls* cKO groups. The original RamDA-seq paper carefully addresses batch effects and demonstrates that the method is highly precise, with minimal batch effect influence [19].

As requested, we have included UMAP and PCA plots from the merged datasets to assess the extent of batch effects (S4 Fig). Potential batch effects during library preparation were negligible based on UMAP clustering analysis using exogenous ERCC RNA spike-in transcripts, which showed a highly mixed genotype-independent distribution (S4A Fig). In addition, PCA for whole cells showed that the major PC axes were not associated with batch (genotype) (S4B Fig), although detailed exploration found minor possible batch effects around PC4 and PC5. These data support our data integration without normalization for batch effects to avoid loss of biological information as mentioned above. A description regarding batch effects has been added in L170-176 of the manuscript to further clarify this point.

3. In Fig 3D and 5F, authors should verified the expression of key DEG such as PDLIM4 and RPL27A by immunostaining in Wls cKO and control ovary at PD7 mice.

**Response 3-3.** Thank you for your suggestion. We have performed the requested immunostaining and updated the manuscript accordingly. We replaced Fig 3G with new immunostaining images that confirm PDLIM4 expression differences in *Wls* cKO and control ovaries at PD7. The updated figure shows differential expression patterns in GCs from secondary follicles, consistent with our RNA-seq data. Besides, we added new immunostaining data for RPL27A in Fig 5H, which aligns with the RNA-seq results, showing high expression in primordial follicles and low expression in growing follicles in oocytes. This expression pattern is consistent between *Wls* cKO and control groups. The relevant sections of the manuscript have been updated to reflect these changes.

4. In Fig 6D, authors should verified the expression and statistical results of NR1H2 and ENO1 by RT PCR in wild type and Wls cKO ovary.

**Response 3-4.** We performed qPCR analysis using RT-RamDA cDNA from the sequenced plates, focusing on oocytes, which we had previously reported to be classified into three subclusters (O1: Dormant, O2: Activated-Ctrl, and O3: Activated-cKO). Given that qPCR measures absolute RNA levels, we recognized that variations in RNA quantity associated with oocyte maturation could potentially skew the results. To account for this, we normalized the data using FSC-Area, representing cell size, to reflect relative RNA levels, similar to what was observed in our scRNA-seq data. This normalization allowed us to replicate the scRNA-seq findings, demonstrating that the expression levels of *Nr1h2* and *Eno1* are indeed elevated in O3. We have included this data as S5C and S5D Fig in the revised manuscript and have included explanation in the main text at L 420-421.

**Reviewer #4**: Elucidation of the role and molecular mechanism of WNT-driven pathways in GC maturation, primordial follicle activation and folliculogenesis is important for understanding ovarian physiology and female reproductive biology. Whilst interesting, there are several concerns that need to be addressed or clarified.

1. Line 541-575, in the”single cells sorting”, how authors to ensure only GCs were separated from the follicles? and what method was utilized to further identify the cells that were used for transcritomic analysis?

**Response 4-1.** We apologize if the manuscript did not clearly communicate the methods used for cell sorting and identification. Contrary to the interpretation that only GCs were isolated, our study focused on separating ovarian somatic cells, which were labeled using a genetically modified mouse expressing tdTomato under the control of the SF1 promoter. This labeling allowed us to specifically mark SF1-positive somatic cells, including GCs, theca cells, ovarian epithelial cells, and other somatic cell types. Additionally, we utilized immunostaining to identify oocytes by targeting KIT, a known marker of these cells. For the subsequent transcriptomic analysis, we employed well-established and widely recognized marker genes to identify and categorize the isolated cell types. Specifically, marker genes such as *Amh* and *Inhbb* were used to identify GCs. This approach ensured the accurate classification of the sorted cells, allowing us to analyze the transcriptomic profiles specific to each cell type. We trust this explanation addresses your concerns.

2. Line 166-172, Line 577-586, in the “Quality control” of scRNA-seq, principal component analysis (PCA) related results that can demonstrated the most important correlation or repeatability between the “single cells” samples should be supplemented.

**Response 4-2.** In response to your comment, we have supplemented our manuscript with additional PCA results to further demonstrate the correlation and repeatability between the single-cell samples. Specifically, we have incorporated figures that provide a clear visualization of how the single cells cluster based on key quality control metrics, including genome alignment rate, mitochondrial gene content, and rRNA percentage (S3 Fig). These PCA plots highlight the separation of low-quality samples and confirm the robustness of our dataset. See also the response to the assessment of batch effect (Response 3-2), which confirmed the repeatability of data from high quality single cells between batches in major PCs (S4 Fig).

3. To facilitate readers to understand the critical role of the WNT-signaling pathway and its molecular signature in regulating normal follicular development, the newly revealed key genes or signaling pathways involved in crosstalk with GCs, GC maturation and differentiation, and oocyte growth should be clearly indicated or provided, and one or two of them are suggested to be further verified.

**Response 4-3.** Thank you for your suggestion. In response, we conducted qPCR experiments focusing on the candidate genes *Fosb*, *Bmp2*, and *Tsc22d1*, which are potentially influenced by WNT signaling in GCs, as indicated by the data in Fig 4C and 4D. Using RT-RamDA cDNA derived from the same samples as those used in our scRNA-seq analysis, we assessed the expression levels of these genes in GCs (S5A and S5B Fig). Our qPCR results confirm the differential expression between GCs from *Wls* cKO (G4, G5, and G6) and control groups (G2, G3). These findings have been incorporated into the revised manuscript at L315-320.
